# A difficult case of Austrian syndrome: a case report

**DOI:** 10.1186/s43044-025-00676-6

**Published:** 2025-08-12

**Authors:** Sonia Peribáñez, Iván De María-Mier, Diana Batin, Mario Martínez-Fleta, Marta Antonio-Martín, Carmen Aured-Guallar, José M. Vallejo-Gil, Alexander S. Vaca-Núñez, Rosa M. Martínez-Álvarez, Ruth Caballero-Asensio

**Affiliations:** 1 Department of Cardiology, Hospital Universitario Miguel Servet, Zaragoza, Spain; 2 Department of Cardiac Surgery, Hospital Universitario Miguel Servet, Zaragoza, Spain; 3 Department of Infectious Diseases, Hospital Universitario Miguel Servet, Zaragoza, Spain

**Keywords:** Case report, Austrian syndrome, Pneumococcus, Endocarditis, Meningitis

## Abstract

**Background:**

The Austrian syndrome is a rare but malignant triad consisting of pneumonia, meningitis, and endocarditis caused by an invasive pneumococcal infection, with a mortality rate of approximately 32%, rising to over 60% if not diagnosed early. Most of the knowledge about this rare disease comes from case reports. The uniqueness of this case lies in the late presentation of endocarditis.

**Case presentation:**

A 59-year-old woman with a medical history of hypertension, dyslipidemia, hypothyroidism, and mesangial proliferative glomerulonephritis was admitted to our hospital with meningitis and pneumonia with bacteremia caused by *Streptococcus pneumoniae.* After receiving antibiotic treatment, the patient improved, and an echocardiogram was performed, ruling out endocarditis. She was discharged and readmitted three weeks later due to endocarditis with an acute perforation of the aortic valve, which required urgent surgery. Fortunately, the patient survived.

**Conclusion:**

In cases of invasive pneumococcal disease with involvement of more than one focus, the possibility of developing infective endocarditis should be considered, especially in cases of hemodynamic instability or heart failure. The Austrian syndrome is a triad that should not be overlooked due to its high mortality rate, especially the possibility of the late onset of endocarditis.

## Background

The Austrian syndrome is a triad consisting of pneumonia, meningitis, and endocarditis caused by an invasive pneumococcal infection. It was first described by William Osler in the nineteenth century. Dr. Austrian revisited some of the diagnostic criteria and the triad of endocarditis, pneumonia, and meningitis then took his name [1]. Since then, there have not been many advances in the understanding of this rare pathology. Most of the knowledge we have about it comes from case reports [2].

Its estimated incidence is around 0.9–7.8 cases per ten million inhabitants per year, with a mortality rate of approximately 32%, rising to over 60% if not diagnosed early. The main cause of death is endocarditis [3]. In the current antibiotic and vaccine era, its incidence is progressively decreasing [4]. However, it remains a condition; we must always keep in mind due to the fatal outcome associated with delayed diagnosis [4, 5].

The peculiarity of this case lies in the late onset of endocarditis, which occurred five weeks after meningitis and pneumonia, and 3 weeks after discharge, following a negative initial evaluation and completion of 2 weeks of therapy, making it a significant clinical challenge. The aim of this case report is to expand knowledge about this pathology, as well as to alert the scientific community about a possible late presentation of endocarditis.

## Case presentation

A 59-year-old woman with a medical history of hypertension, dyslipidemia, hypothyroidism, and mesangial proliferative glomerulonephritis—diagnosed 18 years ago, currently stable and not requiring immunosuppressive therapy—presented to the emergency department with a 2-day history of fever, chills, progressively worsening headache unresponsive to conventional analgesics, and sudden bilateral deafness.

Upon arrival at the emergency department, the patient was hemodynamically stable, with a temperature of 37 °C (98.6 ºF). Physical examination revealed a preserved level of consciousness but with temporal and spatial disorientation, moderate neck stiffness, and complete bilateral deafness.

Initial labortory investigations revealed leukocytosis with a white blood cell count of 13.5 × 10^3^ cells/mm^3^ and neutrophilia of 11.9 × 10^3^ cells/mm^3^, along with elevated C-reactive protein (CRP) at 25.02 mg/dL (normal value < 0.5 mg/dL) and procalcitonin at 1.92 ng/mL (normal value < 0.5 ng/mL).

Computed tomography (CT) scan of the head did not reveal any acute abnormalities, nor did the portable chest X-ray. A lumbar puncture was performed, yielding cloudy fluid. With an initial diagnosis of meningitis, treatment was immediately initiated with 2 g of ceftriaxone twice a day, 1000 mg of vancomycin three times a day, 2 g of ampicillin every 4 h, and 8 mg of dexamethasone every 8 h.

The analysis of the cerebrospinal fluid (CSF) showed an increase in red blood cell count (1700 cells/mm^3^), white blood cells (190 cells/mm^3^) with a predominance of neutrophils (85%), elevated protein levels (121 g/L), and decreased glucose levels (6.3 g/L).

Pneumococcal antigen in urine was negative. After 48 h, *S. pneumoniae* serotype 23B was isolated from blood cultures, with a penicillin MIC of 0.25 µg/mL and a ceftriaxone MIC < 0.25 µg/mL, allowing de-escalation of antibiotic therapy to monotherapy with ceftriaxone. The CSF culture was negative, but polymerase chain reaction (PCR) was positive for *S. pneumoniae.* The patient had not been previously vaccinated against pneumococcus.

During the hospital admission, a follow-up chest X-ray performed on day 11 due to the onset of productive cough and revealed a left basal consolidation with an infectious appearance that was not previously present.

The patient developed edema in the lower extremities, without any other clinical signs suggestive of heart failure or endocarditis. Therefore, a transthoracic echocardiogram was performed, which showed no signs of endocarditis, and the symptom was attributed to her previous renal condition, with subsequent resolution before discharge.

Finally, after receiving targeted antibiotic treatment for 2 weeks, with clinical improvement, normalization of laboratory findings, and resolution of the pulmonary consolidation, the patient was discharged.

**Fig. 1:**
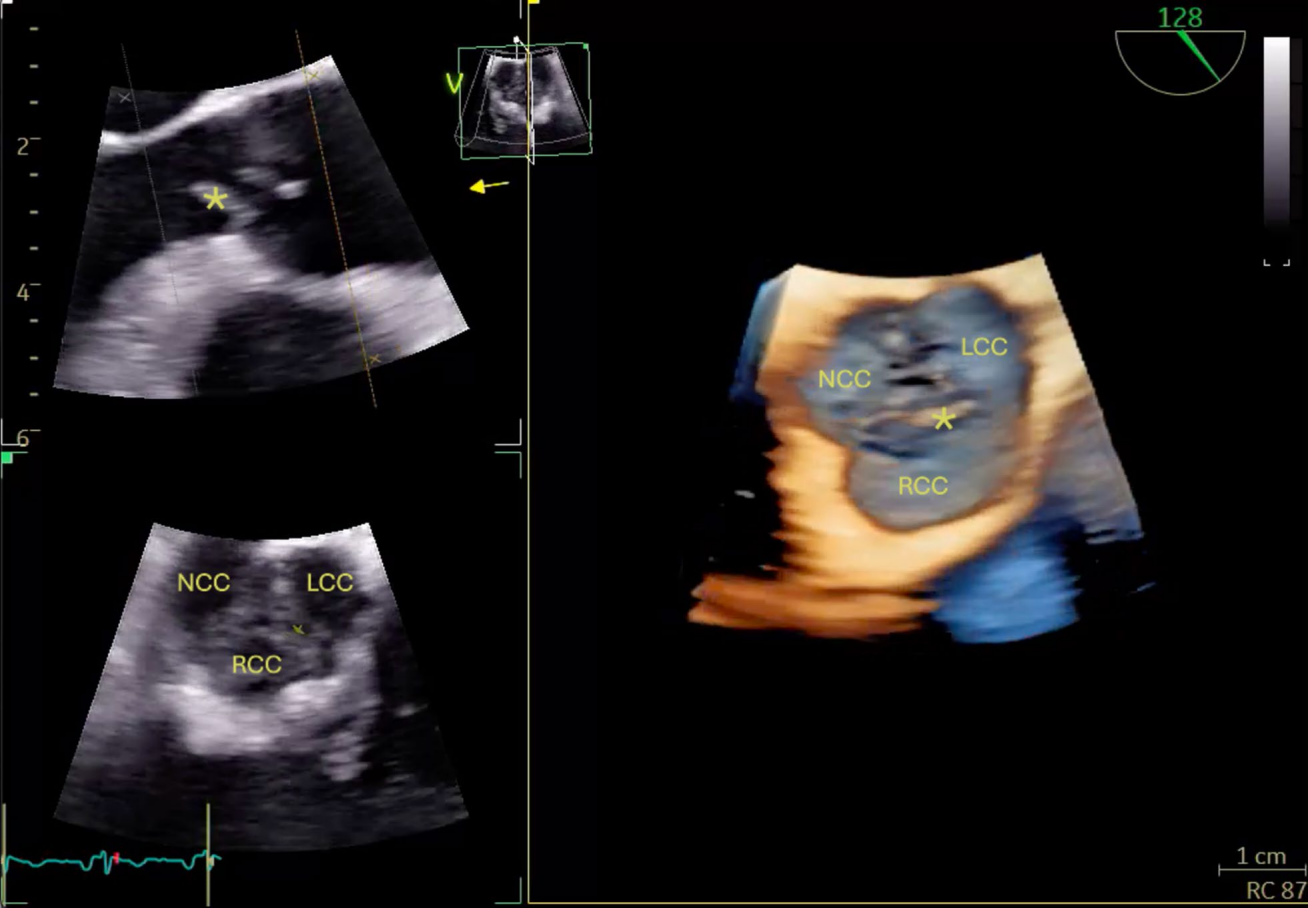
Right coronary cusp disorganized and with a serpiginous vegetative mass approximately 13–14 mm in size, prolapsing extensively into the left ventricular outflow tract (LVOT). Additional small mobile images are observed on the aortic side of the right coronary cusp. RCC: right coronary cusp; LCC: left coronary cusp; NCC: non-coronary cusp. The large vegetation is marked with an asterisk (*)

**Fig. 2:**
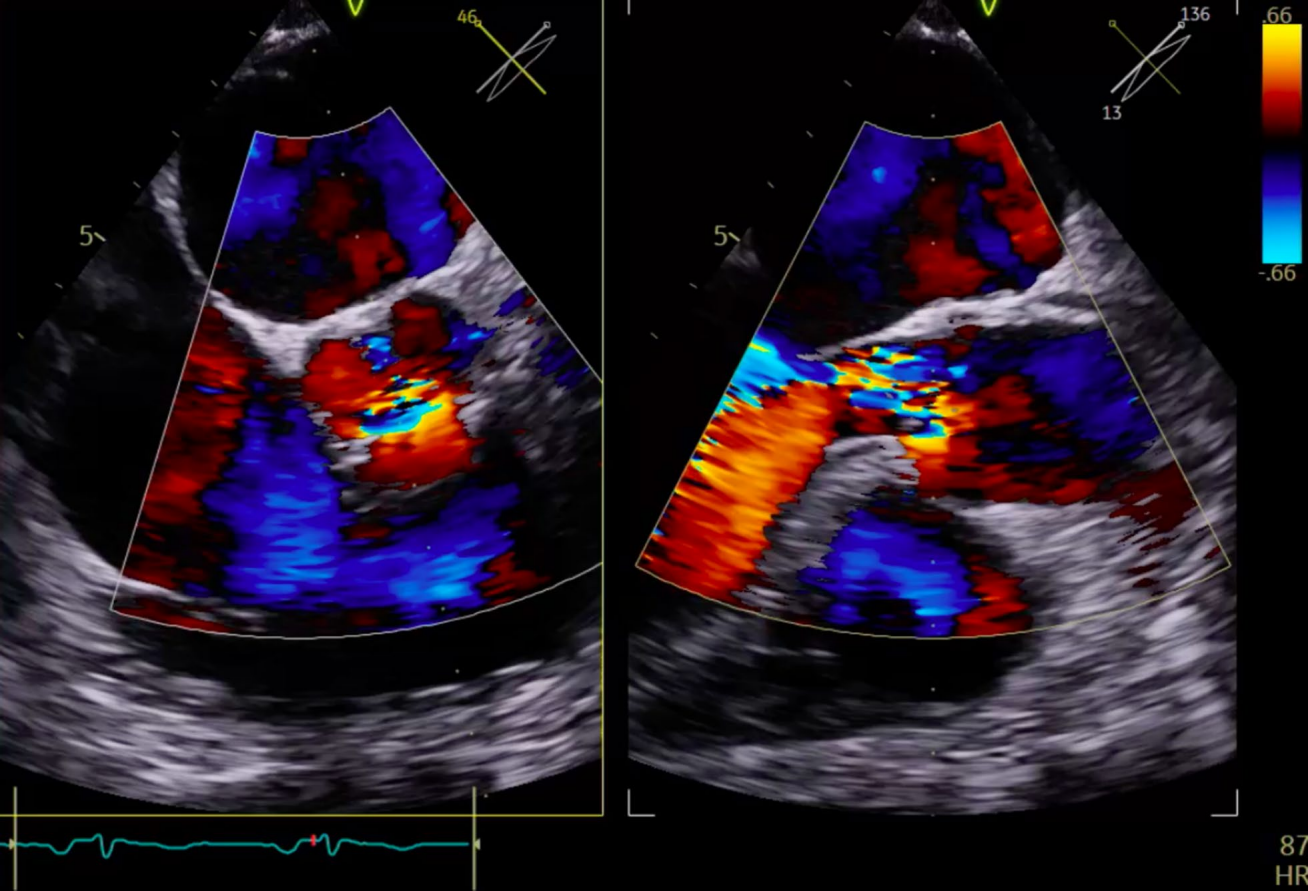
Severe eccentric aortic insufficiency (AI) directed toward the anterior leaflet of the mitral valve (MV)

Three weeks after discharge, 5 weeks after the onset of the meningitis symptoms, the patient returned to the emergency department with high fever, chills, and general malaise. She was hemodynamically stable, with normal oxygen saturation. Physical examination revealed a cardiac murmur. A CT scan and a lumbar puncture were performed, both with normal results. A transthoracic echocardiogram (TTE), followed by a transesophageal echocardiogram (TEE), revealed a 14 mm vegetation on the right coronary cusp, along with other smaller vegetations (Fig. 1). The right coronary cusp was perforated, leading to severe aortic regurgitation (Fig. 2). The other valves showed no signs of endocarditic involvement.

The patient underwent urgent cardiac surgery, with an aortic valve replacement using a mechanical prosthesis. Given the suspicion of pneumococcal involvement, initial treatment was prescribed with ceftriaxone, gentamicin, and daptomycin. The rationale for administering daptomycin was to cover a potential *Staphylococcus aureus* infection, considering the delayed presentation of what appeared to be resolved pneumococcal meningitis and pneumonia, while reducing the risk of nephrotoxicity. Blood and valve cultures were negative, but 2 weeks later, the valve PCR was positive for *S. pneumoniae,* leading to the discontinuation of daptomycin. The delay in the result was due to the samples being sent to a reference center for 16S rRNA testing, a molecular sequencing test.

The triad of endocarditis, meningitis, and pneumonia caused by this pathogen confirmed the diagnosis of Austrian syndrome. Given the previous isolation of *S. pneumoniae* with intermediate resistance to penicillin, the patient completed 4 weeks of ceftriaxone and 2 weeks of gentamicin.

The patient survived this unfortunate triad, with hearing loss as the only sequela. A year and a half later, the patient feels well, exercises regularly, and has a good quality of life.

## Discussion

*Streptococcus pneumoniae* is a Gram-positive encapsulated bacterial pathogen [1, 6]. It is a common commensal of the nasopharynx in healthy humans. The estimated prevalence of colonization in adults is less than 10%, while in children, it ranges from 27 to 65%. The microorganism is transmitted between people through droplets [7].

*Streptococcus pneumoniae* is an opportunistic pathogen that typically behaves as a quiescent colonizer, but it is well-known that it can cause a wide range of diseases in children, the elderly, and immunocompromised individuals [6]. Pneumococcus typically causes infections by spreading to adjacent areas, such as in otitis or airway infections. When it spreads to a normally sterile area (blood, pleural fluid, or CSF), it results in a group of infections classified under the term invasive pneumococcal disease (IPD), which includes a large proportion of community-acquired pneumonia and meningitis [8]. However, the incidence of endocarditis is very low, occurring in only 1.2% (0.8–1.6) of patients with bacteremia caused by this pathogen [9].

In a recent systematic review that includes 69 case reports of Austrian syndrome, conducted by Madu et al. [2], the mean age was 56.5 years, with a male-to-female ratio of 2.4:1. The prevalence of alcoholism was 41%. Other risk factors that have been described include diabetes mellitus, chronic kidney disease, liver and pulmonary disease, asplenia, and other forms of immunosuppression [1].

In the previously mentioned review, the most common symptoms at the time of presentation were altered mental status (69%) and fever (65%). A cardiac murmur was detected in 78% of patients, highlighting the importance of the physical examination [2]. In another review conducted by Kanakadandi et al. [10] on Austrian syndrome, 53% of patients exhibited symptoms of heart failure.

In Austrian syndrome, endocarditis primarily affects the native valves on the left side of the heart. The aortic valve is most frequently involved (49.32%), followed by the mitral valve (28.77%) and combined involvement of both (28.77%). The tricuspid or pulmonary valves are involved in less than 5% of cases. Valvular regurgitation is severe in 70.18% of cases, moderate in 19.3%, and mild in 10.50% [1, 10].

The initial symptoms of endocarditis are often nonspecific, and the diagnosis may be delayed in the absence of classic signs of endocarditis in two out of three patients, with the diagnosis often made only when significant valvular insufficiency is present [1]. In patients with pneumococcal meningitis who are hemodynamically unstable or with involvement of more than one focus, a TEE should be considered in order to verify or rule out the presence of endocarditis [11].

According to the review by Madu et al. [2], nearly 50% of patients already had a murmur at admission, and the progression of symptoms until diagnosis ranged from 1 to 21 days, with a median of 8, which is much shorter than in our patient, whose clinical signs of endocarditis appeared 5 weeks after the onset of meningitis and three weeks after completing antibiotic treatment.

Microbiological diagnosis is based on blood cultures obtained before the initiation of antibiotic treatment. Blood cultures were positive in all cases, whereas CSF cultures were positive in 76% of cases [11]. In another review, blood cultures were negative in 31% of the cases, most of which were obtained after the initiation of antibiotic treatment [3].

The antibiotic treatment recommended in the *2023 ESC Guidelines for the management of endocarditis* caused by *S. pneumoniae* with associated meningitis is either ceftriaxone or cefotaxime, alone or combined with vancomycin, depending on the antibiotic sensitivity pattern. In cases of increased susceptibility or resistance to penicillin, it is recommended to add gentamicin for 2 weeks [12]. In the review by Madu et al. [2], the average duration of antibiotic treatment was 5.6 weeks. The indications for cardiac surgery are summarized in the previous guidelines. The main indication is heart failure, followed by persistent infection and peripheral emboli [12]. Two-thirds of patients require cardiac surgery, with valve replacement being the most common surgical intervention [2].

## Conclusions

In conclusion, Austrian syndrome is an infrequent triad consisting of pneumonia, meningitis, and endocarditis caused by *S. pneumoniae*. Clinical suspicion and physical examination are key points for achieving an early diagnosis and improving the survival of these patients. The peculiarity of this case lies in the late cardiac presentation, which, according to published case series, usually occurs in the 1st week. Thanks to antibiotic treatment and vaccination, it is expected that its incidence will be even less frequent in the coming decades, but it should not be forgotten due to the high morbidity and mortality associated with it.

## Data Availability

No datasets were generated or analysed during the current study.

## Abbreviations

CRP: C-reactive protein
CSF: Cerebrospinal fluid
CT: Computed tomography
GCS: Glasgow Coma Scale
IPD: Invasive pneumococcal disease
LVOT: Left ventricular outflow tract
MRI: Magnetic resonance imaging
PCR: Polymerase chain reaction
TEE: Transesophageal echocardiogram
TTE: Transthoracic echocardiogram

## References

[1] Pandey N, Khan Y, Okobi T, Uhomoibhi D, Abolurin A, Akinlabi OA et al (2023) Austrian syndrome: the forgotten triad of a complex condition in an antibiotic era. Cureus 15(2):c99. 10.7759/cureus.32106 36751572 10.7759/cureus.c99 PMC9898581

[2] Madu A, Alex-Okoro T, Okoduwa A, Cotton J (2024) Austrian syndrome: report of one case and a systematic review of case reports—new insights. Clin Med 24(3):100205. 10.1016/j.clinme.2024.100205 10.1016/j.clinme.2024.100205 PMC11109293 38649138

[3] Rodríguez Nogué M, Gómez Arraiz I, Ara Martín G, Fraj Valle MM, Gómez Peligros A (2019) Austrian syndrome: A rare manifestation of invasive pneumococcal disease. A case report and bibliographic review. Rev Espanola Quimioter 32(2):98–113 PMC6441982 30880376

[4] Shin YI, Papyan N, Cedeño H, Stratidis J (2020) Austrian syndrome: the deadly triad. IDCases 22:e00948. 10.1016/j.idcr.2020.e00948 32923368 10.1016/j.idcr.2020.e00948 PMC7473259

[5] Coronel BI, Vasco D, Silva JFC, Rodríguez C, Vela MTA (2020) Síndrome de Austrian: quien no sabe lo que busca, no entiende lo que encuentra. Rev Colomb Cardiol 27(5):473–476. 10.1016/j.rccar.2019.11.006

[6] Li L, Ma J, Yu Z, Li M, Zhang W, Sun H (2023) Epidemiological characteristics and antibiotic resistance mechanisms of Streptococcus pneumoniae: an updated review. Microbiol Res 266:127221. 10.1016/j.micres.2022.127221 36244081 10.1016/j.micres.2022.127221

[7] Henriques-Normark B, Tuomanen EI (2013) The pneumococcus: epidemiology, microbiology, and pathogenesis. Cold Spring Harb Perspect Med 3(7):a010215. 10.1101/cshperspect.a010215 23818515 10.1101/cshperspect.a010215 PMC3685878

[8] Serrano-Heranz R, Sicilia-Urbán JJ, Sanz-Rojas P (2010) Infecciones por neumococo. Clasificación. Factores predisponentes. Aspectos patogénicos de relevancia clínica o diagnóstica. Manifestaciones clínicas. Formas de comienzo. Medicine 10(50):3352–3359. 10.1016/S0304-5412(10)70042-1 32287886 10.1016/S0304-5412(10)70042-1 PMC7143697

[9] Chamat-Hedemand S, Dahl A, Østergaard L, Arpi M, Fosbøl E, Boel J et al (2020) Prevalence of infective endocarditis in streptococcal bloodstream infections is dependent on streptococcal species. Circulation 142(8):720–730. 10.1161/CIRCULATIONAHA.120.046723 32580572 10.1161/CIRCULATIONAHA.120.046723

[10] Kanakadandi V, Annapureddy N, Agarwal SK, Sabharwal MS, Ammakkanavar N, Simoes P et al (2013) The Austrian syndrome: a case report and review of the literature. Infection 41(3):695–700. 10.1007/s15010-012-0361-3 23124908 10.1007/s15010-012-0361-3

[11] Poulsen JB, Moser C, Espersen K, Moller K (2011) Austrian syndrome. Case Rep 2011:0920103368. 10.1136/bcr.09.2010.3368 10.1136/bcr.09.2010.3368 PMC3062056 22715253

[12] Delgado V, Ajmone Marsan N, De Waha S, Bonaros N, Brida M, Burri H et al (2023) 2023 ESC guidelines for the management of endocarditis. Eur Heart J 44(39):3948–4042. 10.1093/eurheartj/ehad193 37622656 10.1093/eurheartj/ehad193

